# In search of disorders: internalizing symptom networks in a large clinical sample

**DOI:** 10.1111/jcpp.13044

**Published:** 2019-03-21

**Authors:** Eoin McElroy, Praveetha Patalay

**Affiliations:** ^1^ Institute of Psychology, Health and Society University of Liverpool Liverpool UK

**Keywords:** Nosology, depression, anxiety, comorbidity, developmental psychopathology, network analysis

## Abstract

**Background:**

The co‐occurrence of internalizing disorders is a common form of psychiatric comorbidity, raising questions about the boundaries between these diagnostic categories. We employ network psychometrics in order to: (a) determine whether internalizing symptoms cluster in a manner reflecting DSM diagnostic criteria, (b) gauge how distinct these diagnostic clusters are and (c) examine whether this network structure changes from childhood to early and then late adolescence.

**Method:**

Symptom‐level data were obtained for service users in publicly funded mental health services in England between 2011 and 2015 (*N* = 37,162). A symptom network (i.e. Gaussian graphical model) was estimated, and a community detection algorithm was used to explore the clustering of symptoms.

**Results:**

The estimated network was densely connected and characterized by a multitude of weak associations between symptoms. Six communities of symptoms were identified; however, they were weakly demarcated. Two of these communities corresponded to social phobia and panic disorder, and four did not clearly correspond with DSM diagnostic categories. The network structure was largely consistent by sex and across three age groups (8–11, 12–14 and 15–18 years). Symptom connectivity in the two older age groups was significantly greater compared to the youngest group and there were differences in centrality across the age groups, highlighting the age‐specific relevance of certain symptoms.

**Conclusions:**

These findings clearly demonstrate the interconnected nature of internalizing symptoms, challenging the view that such pathology takes the form of distinct disorders.

## Introduction

Internalizing disorders (e.g. depression, anxiety, phobias) are amongst the most common forms of psychopathology (Kessler et al., 2005; Moffitt et al., 2007; Ormel et al., 1994; Polanczyk, Salum, Sugaya, Caye, & Rohde, 2015), and globally they are a leading cause of nonfatal disease burden (Ferrari et al., 2013; Whiteford et al., 2013). Comorbidity rates for these disorders are typically estimated at 40%–60% (Essau, 2008; Essau, Lewinsohn, Lim, Moon‐ho, & Rohde, 2018; Kessler et al., 2005), and the recent DSM‐V field trials highlight the poor inter‐rater reliability of these diagnostic categories (Regier et al., 2013). This overlap may be due, at least in part, to the manner in which we have conceptualized and measured psychopathology. Nosologies such as the DSM and ICD characterize mental health problems as a set of discrete, ‘disease‐like’ entities. Although this approach has undoubtedly led to advancements in our understanding of mental ill‐health, limitations such as arbitrary thresholds (Bebbington, 2015; Krueger & Eaton, 2015), heterogeneity within diagnostic categories (Fried, 2015; Olbert, Gala, & Tupler, 2014) and symptom overlap across diagnostic categories (Borsboom, Cramer, Schmittmann, Epskamp, & Waldorp, 2011), have likely contributed to the problems of high comorbidity and poor reliability. These issues in turn may have impeded our attempts to uncover and understand core physiological markers (Cross‐Disorder Group of the Psychiatric Genomics Consortium, 2013; Kapur, Phillips, & Insel, 2012; Kendler, 2005; Sullivan, Daly, & O'donovan, 2012) and environmental risk factors for psychopathology (Green et al., 2010), and this has led to increasing calls to move towards data‐driven models that may better capture the inherent complexity of psychopathological phenotypes (Kotov, Krueger, & Watson, 2018; Van Dam et al., 2017).

The network perspective is a data‐driven approach that has gained considerable momentum in recent years (Borsboom, 2017). Rather than focus on underlying latent/disease‐like entities, it conceptualizes psychopathology as a complex network of directly associated, often reinforcing symptoms. Risk factors (e.g. genetic disposition, environmental stressors) are proposed to activate individual symptoms, which in turn trigger additional symptoms, initiating a cascade of effects that may eventually settle into a state of mutual reinforcement, even after the removal of the initial stressor(s) (Borsboom, 2017). Even though the majority of network analyses to date has been cross‐sectional, and therefore cannot support the causal interpretations that are central to network theory, cross‐sectional networks remain a useful means of exploring patterns of comorbidity across individuals (Bos et al., 2017). Under this interpretation, what may be considered ‘disorders’ are groups or clusters of symptoms that are strongly associated with one another. This focus on individual symptoms is the main advantage of the network approach; it allows us to quantify the importance of each symptom within the context of the overall network, whilst also enabling us to observe how and where symptoms are related to one another. As such, the network approach may provide a more detailed and nuanced description of the structure of psychopathology, which in turn may help us discern how distinct our diagnostic categories are, and how/where they overlap.

A plethora of recent studies have used network techniques to explore the structure of psychopathological constructs; however, the majority of such studies has focussed their enquiries on single disorder domains (McNally et al., 2015; Robinaugh, LeBlanc, Vuletich, & McNally, 2014) or on a small number of related disorders (Beard et al., 2016; McElroy, Fearon, Belsky, Fonagy, & Patalay, 2018). Given that diagnostic overlap is not limited to a narrow range of disorders, studies of broader symptom networks will help determine the validity of our current disorder categories. However, to our knowledge only three empirical studies have explored symptom networks at the broader spectrum level, for example internalizing and externalizing (Boschloo, Schoevers, van Borkulo, Borsboom, & Oldehinkel, 2016; Boschloo et al., 2015; Goekoop & Goekoop, 2014). All three investigations were in adult samples and reported densely connected symptom networks, with frequent and strong associations within and across traditional diagnostic constructs, which suggests that our diagnostic boundaries are not as well‐defined as previously thought (Boschloo et al., 2015, 2016).

We use network analysis to explore the structure and distinctness of the internalizing spectrum of disorders. Our study builds on previous investigations in several important ways. First, we improve considerably on the statistical power of previous network analyses (Boschloo et al., 2015, 2016; Goekoop & Goekoop, 2014). Network models generally involve the estimation of a large number of parameters, and this number increases exponentially with each additional symptom variable. It has therefore been suggested that many network studies are underpowered due to their reliance on samples that are typically small‐to‐modest in size (Fried & Cramer, 2017). We utilize data from a large clinical sample (*N* = 37,162), which to our knowledge is the most statistically powerful sample to undergo network analysis to date. Second, previous studies have relied on visual inspections of network graphs to determine which symptoms formed distinct disorder clusters (Boschloo et al., 2015, 2016). The present study uses a community detection algorithm (Golino & Epskamp, 2017) to identify community structures (i.e. clusters) within our symptom networks. This will allow us to determine whether symptoms form distinct disorder groupings, and whether these groupings correspond to our most commonly used diagnostic models. Third, few studies have considered symptom networks within a developmental context. This is an important omission, as preliminary evidence suggests that the structure and connectivity of networks may differ across age groups (McElroy, Belsky, Carragher, Fearon, & Patalay, 2018; McElroy, Fearon et al., 2018; Russell, Neill, Carrión, & Weems, 2017), which suggests that certain symptoms and their associations may take on in/decreased relevance over development. Thus, the present study will compare internalizing symptom networks across three different age groups, which may help ascertain: (a) whether diagnostic boundaries become more defined as children age (McElroy, Belsky et al., 2018) and (b) whether and which individual symptoms demonstrate in/decreased relevance as children age. This will shed further light on how internalizing disorders develop with age, and by highlighting developmental relevance of specific symptoms, we may be able to inform age‐tailored assessment/treatment strategies. Finally, from given known sex differences in prevalence of internalizing symptoms in adolescence, we will investigate sex differences in network structure and connectivity.

## Methods

### Participants

This study used routinely collected data from a national best‐practice initiative in the UK between 2011 and 2015 (Fonagy, Pugh, & O'Herlihy, 2017; Wolpert et al., 2016). Information was provided by 81 Child and Adolescent Mental Health Services (CAMHS) operated by the National Health Service, local authorities and voluntary organizations. A total of 38,080 service users provided complete data on a self‐report measure of internalizing psychopathology (The Revised Children's Anxiety and Depression Scale; RCADS; Chorpita, Yim, Moffitt, Umemoto, & Francis, 2000); however, those who were outside the recommended age range for the this measure (i.e. those younger than 8 and older than 18 years) were excluded from further analyses, leaving a total sample of 37,162 clinical cases. This sample was 63% female, with a mean age of 13.63 years (*SD* = 2.37). In order to explore age‐based differences in networks, the sample was divided into three groups: age 8–11 years (*n* = 7,126), 12–14 years (*n* = 14,402) and 15–18 years (*n* = 15,634). The data used in this study are service user records and specific ethical permission was not required to conduct this analysis. Approval was granted by the review board of the institution that hosts the data, the Child Outcomes Research Consortium (CORC; https://www.corc.uk.net/), and all data management and confidentiality protocols governing the use of the dataset were followed.

### Measures

#### The Revised Children's Anxiety and Depression Scale (RCADS)

The RCADS is a 47‐item self‐report measure of internalizing symptoms designed for children/adolescents aged 8–18 years (Chorpita et al., 2000). Symptom frequency is reported on a Likert‐type scale (0 = Never; 1 = Sometimes; 2 = Often; 3 = Always). Items can be summed to form DSM‐based subscale scores corresponding to the following disorders: separation anxiety, social phobia, generalized anxiety, panic, obsessive‐compulsive disorder (OCD) and major depression (Chorpita et al., 2000).

### Analysis

#### Network estimation

Polychoric correlations (available in online supplementary materials) were calculated for the 47 symptom variables, and these were used to estimate and visualize a regularized partial correlation network (i.e. a Gaussian graphical model) using the R package ‘qgraph’ (Epskamp, Cramer, Waldorp, Schmittmann, & Borsboom, 2012). Edges in the network (i.e. lines linking symptoms) can be interpreted similar to partial correlation coefficients, with line thickness reflecting the strength of association between two symptoms after controlling for all other symptoms in the network (Epskamp, Borsboom, & Fried, 2017). In order to reduce the likelihood of type‐I errors, ‘qgraph’ employs the EBICglasso procedure (for details see Epskamp et al., 2017), which shrinks edges and sets very small edges to zero. This produces a sparse network structure that balances parsimony with explanatory power (Epskamp et al., 2017). The EBICglasso procedure was designed to uncover the optimal network structure underlying psychological datasets, which are typically small‐to‐modest in size (Epskamp et al., 2017). However, recent simulation work suggests that this approach may lead to an increase in false‐positives in larger datasets (Williams & Rast, 2018). In order to explore this aspect of our network, we again estimated the overall structure using two newly developed approaches that may offer greater specificity in large samples: thresholded EBICglasso and unregularized model selection (for details see, see http://psychosystems.org/qgraph_1.5). Furthermore, given the large number of nodes in the network, and the similarity of the wording of certain items, we tested whether any nodes could be considered redundant using the Goldbricker function available in the ‘networktools’ package (Jones, 2017), see Appendix S1 for a description.

In order to determine which symptoms were most important within the networks, four commonly used measures of network centrality were examined. Strength was calculated by summing the standardized weights of all significant edges in the network. Nodes (i.e. symptoms) that are high in strength have strong direct association with other nodes in the network (McNally, 2016). Given that strength is calculated based on the absolute value of a given edge (ignoring the sign of the edge), expected influence (Robinaugh, Millner, & McNally, 2016) was calculated using the ‘networktools’ package (Jones, 2017). Expected influence sums the raw weights of edges (+ and ‐), and thus it has been suggested it is a more reliable measure of centrality than strength in networks that contain many negative edges (Robinaugh, Millner, & McNally, 2016). Closeness was calculated by taking the inverse of the sum of the distances of individual nodes from all other nodes. High closeness means a node is highly associated with all other nodes in the network (McNally, 2016). Betweenness was calculated by summing the number of times each node lay on the shortest path between two other nodes. Nodes that are high in betweenness are important for bridging unconnected nodes in a network (McNally, 2016). Network accuracy and centrality stability (i.e. the degree of confidence with which edge weight and centrality rankings can be interpreted) were assessed using the ‘bootnet’ package and the methods outlined by Epskamp et al. (2017). For further description of this process, see the online supplementary materials (Appendix S1).

#### Modularity: investigating diagnostic boundaries

The clustering of symptoms was explored using the walktrap community detection algorithm (Pons & Latapy, 2005), which is available in the 'EGA' package (Golino & Epskamp, 2017). This algorithm is likely to return a clustering solution even in completely random networks; therefore the modularity index *Q* (Newman & Girvan, 2004) was calculated in order to determine how well‐defined this clustering structure was. In practice, most values of *Q* fall between 0.3 and 0.7, with values closer to 0.3 reflecting weakly defined communities, and values around 0.7 reflecting strong community structures (Newman & Girvan, 2004).

#### Developmental and sex differences in network structure and centrality

Developmental and sex differences were explored by splitting the sample by age (three age groups consisting of 8–11; 12–14; 15–18 years) and sex, and estimating separate networks for each group. These networks were compared using the ‘NetworkComparisonTest’ package (vanBorkulo et al., 2016), which tests for structural invariance and invariance in overall connectivity using nonparametric permutation tests (1,000 random permutations were used in this study). For further description of this process, see the online supplementary materials (Appendix S1). In order to ensure that the comparisons were not biased by unequal sample sizes (vanBorkulo et al., 2016), or differences in overall severity between the age groups (Terluin, de Boer, & de Vet, 2016), NCTs were conducted on equal sized groups that were derived via propensity score matching, wherein cases from the two older age groups (*n*_12–14_ = 14,402; *n*_15–18_ = 15,634) were matched to cases from the youngest age group (*n* = 7,126) based on total RCADS score. Cases were matched using the ‘nearest neighbour’ method in the ‘MatchIt’ R package (Ho, Imai, King, & Stuart, 2011). A similar procedure was carried out matching females (*n* = 23,435) to males (*n* = 13,694).

## Results

Descriptive statistics for all 47 RCADS items are presented in the online supplementary materials (Table S1).

### Overall network structure

**Figure 1:**
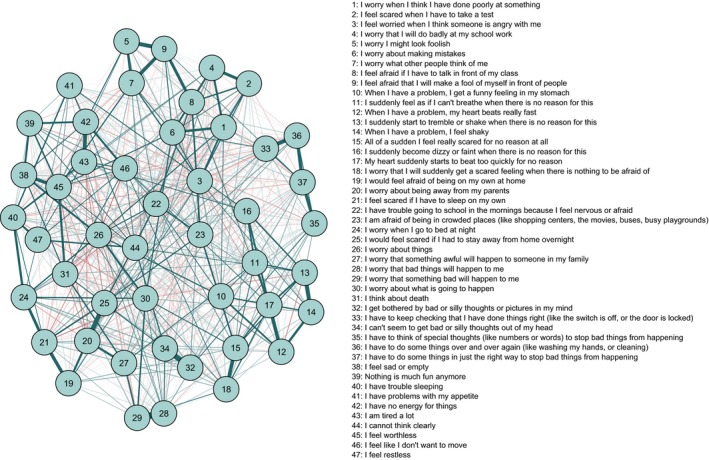
Regularized partial correlation network for the full sample (*N* = 37,162). Teal lines indicate positive association, red lines indicate negative association [Colour figure can be viewed at wileyonlinelibrary.com]

The regularized partial correlation network for the overall sample is presented in Figure 1. Out of a possible 2,162 edges (47*46/2), 688 (32%) were above zero. The network demonstrated excellent accuracy and stability, meaning the rank ordering of edge weights and centrality indices can be interpreted with confidence (Figures S1 and S2). Edge weights ranged from −0.06 to 0.68. Positive edges were more common and stronger (*N* = 387, *M* = 0.07, *SD* = 0.09) than negative edges (*N* = 176, *M* = −0.01, *SD* = 0.01). The strongest edge was between the nodes ‘I worry that bad things will happen to me’ and ‘I worry that something bad will happen to me’. Despite this similarity in wording, the Goldbricker function failed to identify any redundant nodes. This was the only edge weight that was moderate‐to‐strong (i.e. >0.6). Of the other edges, 15 were weak‐to‐moderate (i.e. 0.30–0.59), whereas the rest were weakly associated (i.e. <0.30).

The networks estimated using threshold EBIC and unregularized model selection are presented in the online supplementary materials (Figure S3). When unregularized model selection was used, a slightly less dense network was returned (617 [29%] nonzero edges). In the case of threshold EBIC estimation, a considerable number of the smaller edges were removed from the network (369 [17%] nonzero edges). However, the stronger edges remained largely unaffected by the estimation approach, and therefore the interpretation of the network did not change.

**Figure 2:**
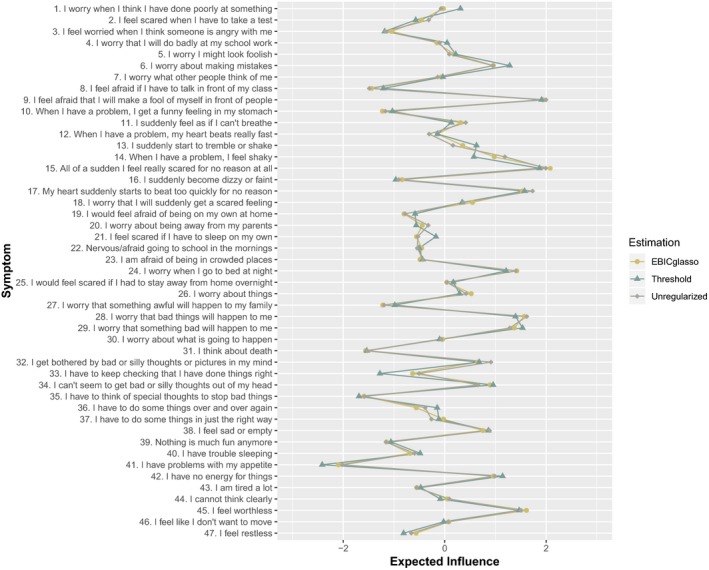
Expected influence values (presented as *Z*‐scores) for full sample (*N* = 37,162) [Colour figure can be viewed at wileyonlinelibrary.com]

Given that the network contained a considerable number of negative edges, our discussion will focus primarily on the expected influence metric, which takes the direction of each edge into account. Expected influence values for the network are presented separately for each estimation method in Figure 2 (strength, betweenness and closeness are the available upon request). The chosen estimation method had little impact on the magnitude and rank ordering of the values, which again demonstrates that the substantive interpretation of the network was not affected by the estimation method.

The symptom with the highest expected influence reflected panic (‘All of a sudden I feel feeling and there is no reason for this’). A fear of making a fool of oneself in public, worry and worthlessness were also high in expected influence. Appetite problems and symptoms of compulsive behaviour had the lowest expected influence.

### Modularity: investigating diagnostic boundaries

**Figure 3:**
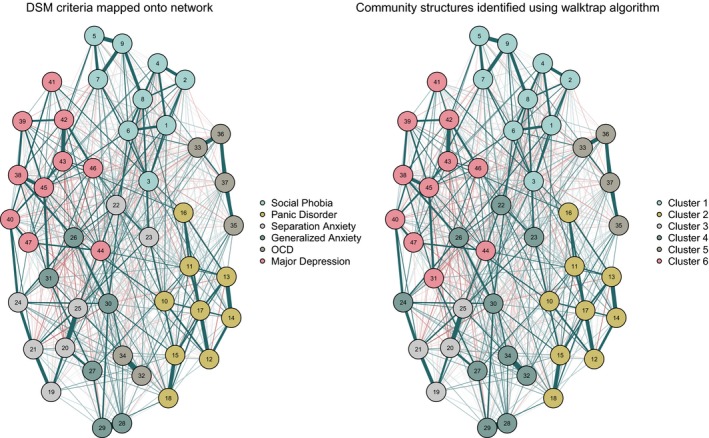
Regularized partial correlation network for full sample (*N* = 37,162). On the left, node colouring reflects the RCADS DSM subscales. On the right, nodes are coloured corresponding to the community structure identified using the walktrap algorithm. Teal lines indicate positive association, red lines indicate negative association [Colour figure can be viewed at wileyonlinelibrary.com]

Based on the walktrap analysis, a community structure of six clusters of nodes had the highest modularity. However, even for this model the *Q‐*index of modularity was low (*Q* = 0.39), indicating the presence of a weak community structure within the data. Figure 3 presents the overall network structure with symptoms coloured according to two different sets of criteria: (a) DSM criteria (RCADS DSM subscales) and (b) communities identified using EGA.

Two of the identified communities perfectly aligned with the RCADS sub‐scales; social phobia and panic disorder. There were some discrepancies between the RCADS and community structures for the remaining symptoms. The major depression subscale of the RCADS was largely identified as a unique community; however, it subsumed the item ‘I think about death’ from the generalized anxiety subscale. Symptoms reflecting compulsions (but not obsessive thoughts) formed a community. The items used to assess obsessive thoughts, along with three pertaining to separation anxiety combined with items from the general anxiety subscale, to form the largest identified community.

### Developmental and sex differences in network modularity, structure and connectivity

Networks were estimated separately for the three age groups (8–11; 12–14; 15–18 years; Figure S4).

Similar to the whole sample, the identified communities were weakly demarcated with no indication of notable clustering. The propensity score matching resulted in equally sized groups, with approximately equal scores (Table S2). Significant differences in the overall structures of the networks were observed between each age group (*M*_(8–11 vs. 12–14) _= 0.15, *p* < .001; *M*_(8–11 vs. 15–18)_ = 0.17, *p* < .001). Approximately 3% of individual edges differed between the youngest and middle age group, and this rose to 8% when comparing the youngest and oldest groups. Global strength (GS) values (i.e. the summed totals of weighted connections) were significantly higher in the two older age groups compared with the youngest age group (∆GS _(8–11 vs. 12–14)_ = 1.33, *p* = .001; ∆GS _(8–11 vs. 15–18)_ = 1.59, *p* < .001). This indicates that older children had more densely connected symptom networks. Ages 12–14 and 15–18 did not differ significantly in global strength.

**Figure 4:**
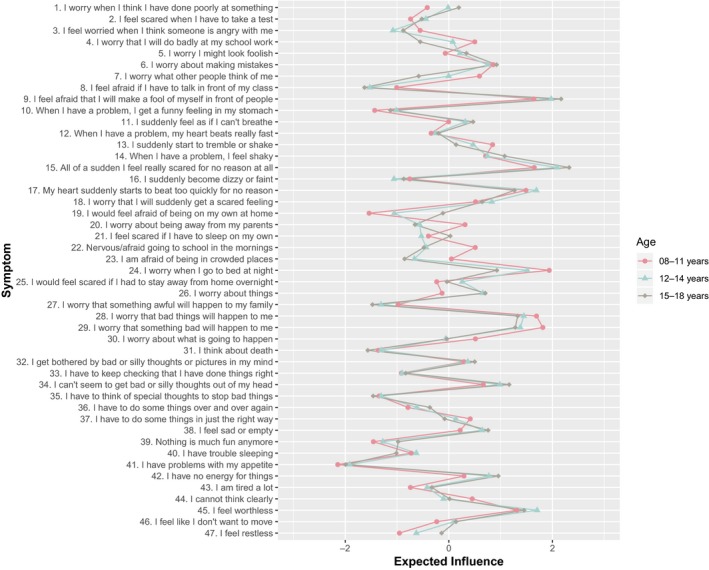
Expected influence values (presented as *Z*‐scores) for different age groups [Colour figure can be viewed at wileyonlinelibrary.com]

Expected influence values for the age‐based networks are presented in Figure 4. Results from bootstrapped 95% difference tests indicated that the rank ordering of strength values were reliable within groups (Figure S5). Although the majority of symptoms demonstrated consistency across age groups, some differences were observed. Although, to our knowledge, it is not yet possible to compare centrality values across groups, we discuss symptoms that differed by approximately 1 standardized *Z*‐score or more. Compared with the youngest group, the oldest age group had higher expected influence values for restlessness, fatigue, general worry and a fear of being away from parents. Compared with the oldest group, those in the youngest group had higher expected influence scores on fears/worries about school, fears going to bed at night and fears about what others think of them.

The networks estimated separately by sex are presented in Figure S6. There was no difference in overall connectivity (∆GS _(m vs. f)_ = 0.45, *p* = .434); however, there was a significant difference in overall structure (*M*_(m vs. f)_ = 0.068, *p* < .001) with approximately 3% of individual edges differing significantly by sex. Centrality values (Figure S7) were also highly comparable across males and females.

## Discussion

This study used network analysis to examine the structure and distinctness of the internalizing spectrum of disorders in a large clinical sample of children and adolescents. Using three different estimation methods, we found a highly interconnected network structure, characterized by a multitude of predominantly weak connections between symptoms. An inspection of network modularity firstly indicated that there was little clustering of symptoms into distinct communities, and in the most differentiated model six communities were weakly demarcated. Moreover, cross‐community associations were widespread, indicating considerable overlap between these symptom groupings. It is thus unsurprising that comorbidity is the rule rather than the exception when strict categorical diagnoses are employed (Kessler et al., 2005; Moffitt et al., 2007; Ormel et al., 1994; Polanczyk et al., 2015). These findings add to the growing body of evidence that challenges the idea of internalizing disorders as distinct disorder entities (Borsboom, 2017; Kotov et al., 2017; McElroy, Belsky, Carragher, Fearon, & Patalay, 2018; McElroy, Fearon et al., 2018).

The networks estimated and the lack of distinct clusters identified in the present study further highlight the interconnected nature of internalizing psychopathology. Taking OCD symptoms as an example, four items pertaining to compulsions formed a community and the two items used to assess obsessions were incorporated into a general anxiety cluster. Cognitive models of OCD posit that dysfunctional beliefs (e.g. threat estimation, control of thoughts, tolerance of uncertainty, perfectionism) lie at the heart of the onset and maintenance of OCD symptoms (Jones, Mair, Riemann, Mugno, & McNally, 2018; McNally, Mair, Mugno, & Riemann, 2017; Tolin, Worhunsky, & Maltby, 2006). However such maladaptive beliefs have been shown to poorly differentiate between those with OCD and other anxiety diagnoses (Tolin et al., 2006; Viar, Bilsky, Armstrong, & Olatunji, 2011), leading some to propose that the obsessive component of OCD is reflective of anxiety (or indeed psychopathology) more generally (Tolin et al., 2006). Collectively the inconsistencies in clustering observed in our data indicate that it is particularly difficult to delineate the most common forms of internalizing psychopathology into clear and distinct disorder categories in childhood and adolescence. This is not to say that the process of aggregating multiple symptoms into disorder‐like constructs cannot be justified. Highly associated groups of symptoms may be conceptually summarized as disorder syndromes. However, the lack of distinct clustering corresponding to our most widely used diagnostic criteria and the high degree of cross‐community associations observed in the present study lend greater support to recent calls for more empirically based conceptualizations of mental illnesses that move away from distinct disorder entities (Kotov et al., 2018).

There were no notable sex differences in the strength and structure of the symptom network, and the community structure identified in the present study remained broadly consistent when the networks were re‐estimated based on different age groups. As such, it does not appear that disorders become more defined or change as children progress from childhood through adolescence (McElroy, Belsky, Carragher, Fearon, & Patalay, 2018; McElroy, Fearon et al., 2018). However, network comparisons revealed that the overall connectivity of networks differed based on age, with more densely connected networks observed in older children. This indicates that, as children develop, the associations between internalizing symptoms increase as a whole, rather than forming increasingly defined clusters of symptoms (McElroy, Belsky, Carragher, Fearon, & Patalay, 2018; McElroy, Fearon et al., 2018). A possible explanation for this increased connectivity is that internalizing symptoms feed into and reinforce one another over time (Borsboom, 2017). This re‐emphasizes the need for early intervention, as recent studies have demonstrated that those with more strongly connected symptom networks are less responsive to treatment (van Borkulo et al., 2015; McElroy, Napoleone, Wolpert, & Patalay, 2019; Schweren, van Borkulo, Fried, & Goodyer, 2018), possibly reflecting maladaptive feedback cycles amongst symptoms that are particularly hard to break.

Along with this increase in the overall strength of associations within the networks, we observed differences in the centrality of specific symptoms across the three age groups. For instance, feelings of restlessness and fatigue were higher in expected influence in the oldest age groups, whereas fears (e.g. going to bed, doing badly at school work) were more central in the youngest group. This suggests a changing expression of symptoms over development, whereby certain symptoms take on in/decreased relevance as children age. It must be noted, however, that a frequently proposed hypothesis, that intervening on highly central symptoms will lead to improved treatment outcomes, has yet to be empirically verified (Fried et al., 2018) and studies that explicitly test this aspect are therefore required.

The main strength of the present study was the statistical power afforded by our large clinical sample. The absence of an edge in any given network indicates one of the two possibilities: (a) the edge does not exist (i.e. the two symptoms are not associated after controlling for all other symptoms in the network) and (b) there is insufficient power for the edge to be detected (Epskamp et al., 2017). Despite this, statistical power remains an under‐researched area of network analysis (Epskamp et al., 2017). Given that the estimated parameters of a network model increase exponentially with each additional symptom variable, it has been suggested that many recent network studies may be underpowered (Fried & Cramer, 2017). The present study improves considerably on the power of previous such network analyses (Boschloo et al., 2016; Goekoop & Goekoop, 2014).

With regard to limitations, the measure used in the present study was shaped by DSM criteria, which are not necessarily reflective of the entirety of emotional/internalizing problems (Fried & Nesse, 2015; Goekoop & Goekoop, 2014), and the in/exclusion of pertinent symptoms can alter the structure of a given network (Fried & Cramer, 2017). In addition, the present study explored the developmental differences by comparing the network structures of three broad age groups rather than longitudinal data from the same participants. Furthermore, although we compared centrality measures across groups, we were unable to test whether such differences were statistically different using currently available software packages. As such, the development methods to compare centrality statistics across groups should be a key priority in network psychometrics. Finally, as with all cross‐sectional networks, which explore group‐level differences, these findings may not generalize to the level of the individual as causality cannot be determined from cross‐sectional data (Bos et al., 2017).

In conclusion, the present study sought to investigate the distinctness of the diagnostic boundaries of internalizing disorders in a large clinical sample of children and adolescents. We found a highly interconnected network structure, comprised of a multitude of relatively weak connections between symptoms. Our data‐driven methods identified a model consisting of six communities; however, given the weak differentiation between these communities, the broader conclusion is that no clear diagnostic boundaries are identifiable in these data. Further analyses in different age groups found that this lack of distinct clustering broadly consistent across childhood and adolescence, with no indication of increased disorder differentiation in older adolescents. However, there were notable difference in the overall importance/centrality of symptoms across these age groups; fears relating to school were found to be more central in younger children, whereas fatigue and restlessness were more central in older children. Overall, these findings challenge the conceptualization of the internalizing spectrum as a set of discrete disorders.

**Key points**

- Internalizing disorders (e.g. depression, anxiety, OCD) are frequently comorbid, raising questions about the boundaries between these diagnostic categories.
- In this network analysis of children presenting to mental health services (*N* = 37,162), we found that internalizing symptoms formed a highly interconnected network structure, with little distinct clustering of symptoms that pertained to DSM diagnostic criteria.
- Symptom networks were broadly consistent across males and females and in different age groups, with symptom connectivity being higher in adolescence than in childhood. Different symptoms were more influential within the networks at different ages indicating developmentally specific experience of internalizing psychopathology.
- This highly interconnected network structure challenges the idea that internalizing disorders are discrete diagnostic entities.

## Supporting information


**Appendix S1.** Methods.
**Table S1.** Item‐level means, standard deviations and 95% confidence intervals.
**Table S2.** Mean RCADS scores pre and post propensity score matching.
**Figure S1.** Results from tests of edge weight accuracy.
**Figure S2.** Results from tests of centrality stability.
**Figure S3.** Networks estimated using alternative methods.
**Figure S4.** Regularized partial correlation networks for the three age groups.
**Figure S5.** Bootstrapped difference tests of strength values by propensity score matched groupings.
**Figure S6.** Networks estimated separately by gender (propensity score matched).
**Figure S7.** Centrality values by gender.
