# Supplementary material for: In search of disorders: internalizing symptom networks in a large clinical sample
Source: J Child Psychol Psychiatry. 2019 Mar 21;60(8):897–906. doi: 10.1111/jcpp.13044 (PMC6767473; doi:10.1111/jcpp.13044)
Supplement: Supplementary file 1 — Appendix S1. Methods. Table S1. Item‐level means, standard deviations and 95% confidence intervals. Table S2. Mean RCADS scores pre and post propensity score matching. Figure S1. Results from tests of edge weight accuracy. Figure S2. Results from tests of centrality stability. Figure S3. Networks estimated using alternative methods. Figure S4. Regularized partial correlation networks for the three age groups. Figure S5. Bootstrapped difference tests of strength values by propensity score matched groupings. Figure S6. Networks estimated separately by gender (propensity score matched). Figure S7. Centrality values by gender. [file JCPP-60-897-s001.docx]

**In search of disorders: Internalizing symptom networks in a large clinical sample**

Eoin McElroy, Praveetha Patalay

**Online Supporting Information**

|  |  |  |
| --- | --- | --- |
| [Appendix S1. Description of edge weight accuracy, centrality stability and network comparison tests](#mS1) | ………………….. | 2. |
| [Table S1. Item-level descriptive statistics](#table1) | ………………….. | 4. |
| [Table S2. Mean RCADS scores pre and post propensity score matching](#tables2) | ………………….. | 6. |
| [Figure S1. Results from tests of edge weight accuracy](#s1) | ………………….. | 7. |
| [Figure S2. Results from tests of centrality stability](#efig2) | ………………….. | 8. |
| [Figure S3. Networks estimated using alternative methods](#s3) | ………………….. | 9. |
| [Figure S4. Regularised partial correlation networks for the three age groups](#S6) | ………………….. | 10. |
| [Figure S5. Bootstrapped difference tests of centrality indices by propensity score matched groupings](#FigS7) | ………………….. | 11. |
| [Figure S6. Networks estimated separately by gender](#S4) | ………………….. | 12. |
| [Figure S7. Centrality values by gender](#S5) | ………………….. | 13. |
|  |  |  |
|  |  |  |

**Appendix S1. Methods**

*Description of edge weight accuracy, centrality stability, Goldbricker method, and network comparison tests*

Edge weight accuracy refers to the degree of confidence with which we can interpret the ranking of the edge weights (strongest to weakest). To assess the accuracy of the networks, bootstrapped 95% confidence intervals (CIs) were calculated for each edge, and the results plotted. A lack of overlap between confidence intervals indicates a significant difference in the strength of two edges (Epskamp et al., 2017). Centrality stability refers to the reliability of the rank ordering of the centrality indices. This was examined using the case-dropping subset bootstrap method (Epskamp et al., 2017); networks were re-estimated using increasingly smaller subsets of the original sample, and correlations between the original centrality indices and the subset centrality indices were calculated. A small-to-moderate decrease in correlation as participants are removed suggests that the order of centrality is relatively stable/reliable. This can be quantified in the form of the correlation stability coefficient, with values above 0.7 deemed to reflect high centrality reliability, and values between 0.25 and 0.7 denoting moderate reliability (Epskamp et al., 2017). The rank ordering of the centrality values was also empirically tested using the methods developed by Epskamp and colleagues (2017); bootstrapped 95% CIs were calculated around the difference scores of each given pair of centrality values, and if these 95% CIs contained zero, the two centrality values were judged to be significantly different. Accuracy and reliability analyses were conducted using the R package ‘bootnet’, based on 1,000 bootstrapped samples (Epskamp et al., 2017).

In any given psychopathological network, two nodes may be measuring the same construct, meaning their inclusion in the network could result in statistical over control. The Goldbricker approach uses a p-value threshold to determine whether a pair of correlations to a third variable are significantly different from each other, and this is repeated for every combination of correlations within a given network in the ‘networktools’ package (Jones, 2017). Different thresholds (ranging 0- 1) can be applied, with values closer to 1 more conservative. For further details, see <https://cran.r-project.org/web/packages/networktools/networktools.pdf>.

The network comparison test (NCT) procedure allows for the comparison of specific edges across networks, and tests invariance in overall connectivity (i.e. global strength) and structure. This procedure is carried out in three phases. First, the two networks in question are estimated and the relevant test statistics are calculated (van Borkulo et al., 2016). For individual edges, the test statistic is the observed difference in edge weight. For invariance in overall connectivity, the test statistic is the difference in global-strength (i.e., difference in sum of edge weights of two networks). For structural invariance, the statistic is the largest individual difference in edge strength observed between the two networks. Second, cases are repeatedly and randomly swapped between networks, and these test statistics re-estimated. Third, a reference distribution is created from these test statistics and statistical significance is determined, with the p-value equal to the proportion of test statistics that have an equal or higher value than the observed test statistic (van Borkulo et al., 2016). Networks were compared using 1,000 random permutations. NCTs were carried out using the ‘NetworkComparisonTest’ package (van Borkulo et al., 2016).

| **Table S1. Item-level means, standard deviations and 95% confidence intervals** | | | | | | | | |
| --- | --- | --- | --- | --- | --- | --- | --- | --- |
|  | Total sample (N=37,162) | | Age 8-11 years  (N=7,126) | | Age 12-14 years  (N=14,402) | | Age 15-18 years (N=15,634) | |
| *Item* | *Mean (SD)* | *95% C.I* | *Mean (SD)* | *95% C.I* | *Mean (SD)* | *95% C.I* | *Mean (SD)* | *95% C.I* |
| I worry about things | 1.97 (0.87) | 1.96-1.98 | 1.60 (0.84) | 1.58-1.62 | 1.94 (0.87) | 1.92-1.95 | 2.18 (0.81) | 2.16-2.19 |
| I feel sad or empty | 1.54 (0.87) | 1.53-1.55 | 1.13 (0.78) | 1.11-1.15 | 1.49 (0.88) | 1.48-1.51 | 1.76 (0.82) | 1.75-1.78 |
| I get a funny feeling in my stomach | 1.43 (1.04) | 1.42-1.44 | 1.18 (1.06) | 1.16-1.21 | 1.40 (1.03) | 1.38-1.42 | 1.57 (1.00) | 1.56-1.59 |
| I worry when I think I have done poorly | 1.70 (1.00) | 1.69-1.71 | 1.41 (1.01) | 1.39-1.44 | 1.63 (0.99) | 1.61-1.65 | 1.90 (0.95) | 1.89-1.92 |
| I would be afraid on my own at home | 0.81 (1.00) | 0.80-0.82 | 1.14 (1.20) | 1.11-1.17 | 0.68 (0.91) | 0.67-0.69 | 0.77 (0.93) | 0.75-0.78 |
| Nothing is much fun anymore | 1.19 (0.93) | 1.18-1.20 | 0.82 (0.86) | 0.80-0.84 | 1.17 (0.92) | 1.15-1.18 | 1.39 (0.90) | 1.38-1.41 |
| I feel scared when I have to take a test | 1.48 (1.08) | 1.47-1.49 | 1.25 (1.10) | 1.22-1.27 | 1.45 (1.08) | 1.43-1.46 | 1.62 (1.04) | 1.61-1.64 |
| I feel worried when someone is angry with me | 1.64 (1.05) | 1.63-1.65 | 1.51 (1.04) | 1.49-1.54 | 1.57 (1.05) | 1.55-1.59 | 1.77 (1.04) | 1.75-1.79 |
| I worry about being away from my parents | 1.04 (1.01) | 1.03-1.05 | 1.51 (1.12) | 1.48-1.54 | 1.00 (0.97) | 0.98-1.01 | 0.86 (0.92) | 0.85-0.88 |
| I get bothered by bad or silly thoughts | 1.47 (1.09) | 1.46-1.49 | 1.25 (1.08) | 1.22-1.27 | 1.40 (1.09) | 1.38-1.42 | 1.65 (1.06) | 1.63-1.66 |
| I have trouble sleeping | 1.59 (1.04) | 1.58-1.60 | 1.45 (1.09) | 1.42-1.47 | 1.53 (1.04) | 1.52-1.55 | 1.70 (1.01) | 1.68-1.71 |
| I worry that I will do badly at school | 1.53 (1.04) | 1.52-1.54 | 1.15 (0.99) | 1.13-1.17 | 1.47 (1.02) | 1.45-1.48 | 1.76 (1.01) | 1.75-1.78 |
| I worry that something will happen to my family | 1.26 (1.04) | 1.25-1.27 | 1.27 (1.07) | 1.25-1.30 | 1.25 (1.03) | 1.24-1.27 | 1.26 (1.03) | 1.24-1.28 |
| I suddenly feel as if I can't breathe | 0.88 (0.97) | 0.87-0.89 | 0.58 (0.86) | 0.56-0.60 | 0.87 (0.97) | 0.86-0.89 | 1.01 (0.99) | 0.99-1.03 |
| I have problems with my appetite | 1.08 (1.03) | 1.07-1.09 | 0.80 (0.99) | 0.78-0.83 | 1.03 (1.02) | 1.02-1.05 | 1.24 (1.02) | 1.23-1.26 |
| I keep checking that I have done things right | 1.17 (1.06) | 1.16-1.18 | 1.03 (1.07) | 1.01-1.06 | 1.15 (1.05) | 1.14-1.17 | 1.25 (1.06) | 1.23-1.26 |
| I feel scared if I have to sleep on my own | 0.56 (0.89) | 0.56-0.57 | 0.86 (1.07) | 0.83-0.88 | 0.49 (0.83) | 0.47-0.50 | 0.50 (0.82) | 0.49-0.51 |
| I feel nervous or afraid going to school | 1.08 (1.09) | 1.07-1.09 | 0.84 (1.02) | 0.81-0.86 | 1.13 (1.10) | 1.11-1.14 | 1.15 (1.09) | 1.13-1.16 |
| I have no energy for things | 1.31 (0.99) | 1.30-1.32 | 0.67 (0.82) | 0.65-0.69 | 1.27 (0.98) | 1.25-1.28 | 1.63 (0.94) | 1.61-1.64 |
| I worry I might look foolish | 1.47 (1.08) | 1.46-1.49 | 1.04 (1.01) | 1.01-1.06 | 1.50 (1.08) | 1.48-1.52 | 1.65 (1.05) | 1.63-1.67 |
| I am tired a lot | 1.75 (0.99) | 1.73-1.76 | 1.18 (0.95) | 1.16-1.20 | 1.71 (0.98) | 1.69-1.72 | 2.04 (0.90) | 2.02-2.05 |
| I worry that bad things will happen to me | 1.35 (1.00) | 1.33-1.36 | 1.17 (0.99) | 1.14-1.19 | 1.30 (1.00) | 1.29-1.32 | 1.47 (0.99) | 1.45-1.48 |
| I can't get bad/silly thoughts out of my head | 1.48 (1.06) | 1.47-1.49 | 1.23 (1.06) | 1.21-1.26 | 1.41 (1.06) | 1.39-1.43 | 1.66 (1.02) | 1.65-1.68 |
| When I have a problem my heart beats fast | 1.45 (1.06) | 1.44-1.46 | 1.16 (1.06) | 1.13-1.18 | 1.41 (1.05) | 1.40-1.43 | 1.63 (1.02) | 1.61-1.64 |
| I cannot think clearly | 1.46 (0.93) | 1.45-1.47 | 1.08 (0.92) | 1.06-1.11 | 1.44 (0.92) | 1.42-1.45 | 1.64 (0.88) | 1.63-1.66 |
| I suddenly start to tremble or shake | 0.88 (0.95) | 0.87-0.89 | 0.59 (0.84) | 0.57-0.61 | 0.87 (0.95) | 0.85-0.89 | 1.02 (0.97) | 1.00-1.04 |
| I worry that something bad will happen to me | 1.20 (0.99) | 1.19-1.21 | 1.10 (0.99) | 1.07-1.12 | 1.17 (0.98) | 1.15-1.19 | 1.28 (0.98) | 1.27-1.30 |
| When I have a problem I feel shaky | 1.22 (1.02) | 1.21-1.23 | 0.91 (0.97) | 0.89-0.93 | 1.20 (1.02) | 1.18-1.21 | 1.37 (1.01) | 1.36-1.39 |
| I feel worthless | 1.36 (1.09) | 1.35-1.37 | 0.81 (0.94) | 0.79-0.84 | 1.34 (1.10) | 1.32-1.35 | 1.64 (1.05) | 1.62-1.65 |
| I worry about making mistakes | 1.63 (1.00) | 1.62-1.64 | 1.28 (0.99) | 1.26-1.31 | 1.59 (1.00) | 1.57-1.61 | 1.82 (0.95) | 1.80-1.83 |
| I have to think special thoughts (numbers/words) | 0.57 (0.89) | 0.56-0.58 | 0.58 (0.90) | 0.56-0.60 | 0.58 (0.89) | 0.56-0.59 | 0.56 (0.88) | 0.55-0.58 |
| I worry what other people think of me | 1.74 (1.10) | 1.73-1.75 | 1.27 (1.06) | 1.25-1.30 | 1.77 (1.11) | 1.75-1.78 | 1.93 (1.05) | 1.92-1.95 |
| I am afraid of being in crowded places | 1.13 (1.12) | 1.12-1.14 | 0.80 (1.04) | 0.78-0.82 | 1.11 (1.12) | 1.09-1.13 | 1.29 (1.12) | 1.27-1.31 |
| All of a sudden I feel really scared | 0.92 (0.96) | 0.91-0.93 | 0.69 (0.89) | 0.67-0.71 | 0.90 (0.96) | 0.89-0.92 | 1.04 (0.98) | 1.02-1.05 |
| I worry about what is going to happen | 1.52 (1.00) | 1.51-1.53 | 1.25 (0.99) | 1.22-1.27 | 1.47 (1.00) | 1.45-1.49 | 1.69 (0.97) | 1.68-1.71 |
| I suddenly become dizzy or faint | 0.79 (0.92) | 0.78-0.80 | 0.44 (0.76) | 0.42-0.46 | 0.81 (0.93) | 0.79-0.82 | 0.93 (0.94) | 0.91-0.94 |
| I think about death | 1.29 (1.04) | 1.28-1.31 | 0.99 (1.02) | 0.97-1.02 | 1.26 (1.04) | 1.24-1.28 | 1.46 (1.01) | 1.45-1.48 |
| I'm afraid of having to talk in front of class | 1.56 (1.17) | 1.55-1.57 | 1.15 (1.10) | 1.13-1.18 | 1.61 (1.17) | 1.59-1.63 | 1.69 (1.16) | 1.67-1.71 |
| My heart suddenly starts to beat too quickly | 0.83 (0.96) | 0.82-0.84 | 0.52 (0.82) | 0.50-0.54 | 0.81 (0.95) | 0.80-0.83 | 0.99 (0.98) | 0.98-1.01 |
| I feel like I don't want to move | 1.14 (0.99) | 1.13-1.15 | 0.78 (0.91) | 0.75-0.80 | 1.11 (0.98) | 1.01-1.13 | 1.34 (0.97) | 1.32-1.35 |
| I worry that I will suddenly get scared | 0.90 (0.95) | 0.89-0.91 | 0.74 (0.89) | 0.72-0.76 | 0.87 (0.94) | 0.86-0.89 | 1.01 (0.98) | 0.99-1.02 |
| I have to do some things over and over again | 0.82 (1.01) | 0.81-0.83 | 0.87 (1.05) | 0.85-0.90 | 0.81 (1.00) | 0.80-0.83 | 0.81 (1.00) | 0.79-0.82 |
| I'm afraid I will make a fool of myself | 1.47 (1.07) | 1.46-1.48 | 1.09 (1.00) | 1.06-1.11 | 1.50 (1.08) | 1.48-1.52 | 1.61 (1.05) | 1.60-1.63 |
| I have to do things in just the right way | 0.83 (0.97) | 0.82-0.84 | 0.81 (0.97) | 0.78-0.83 | 0.84 (0.97) | 0.82-0.85 | 0.83 (0.98) | 0.82-0.85 |
| I worry when I go to bed at night | 1.23 (1.09) | 1.22-1.24 | 1.10 (1.09) | 1.07-1.12 | 1.15 (1.07) | 1.13-1.16 | 1.38 (1.07) | 1.36-1.39 |
| I would feel scared staying away overnight | 0.63 (0.92) | 0.62-0.64 | 0.96 (1.09) | 0.93-0.98 | 0.59 (0.88) | 0.58-0.61 | 0.52 (0.83) | 0.51-0.54 |
| I feel restless | 1.36 (0.99) | 1.35-1.37 | 1.18 (1.05) | 1.15-1.20 | 1.30 (0.99) | 1.28-1.32 | 1.50 (0.95) | 1.49-1.52 |

**Table S2. Mean RCADS scores pre and post propensity score matching**

|  | *Pre metching* |  |  | *Post matching* |  |  |
| --- | --- | --- | --- | --- | --- | --- |
| Group | N | Mean | SD | N | Mean | SD |
| Age 8-11 | 7,126 | 48.21 | 25.84 | 7,126 | 48.21 | 25.84 |
| Age 12-14 | 14,402 | 57.32 | 28.58 | 7,126 | 48.21 | 25.84 |
| Age 15-18 years | 15,634 | 64.77 | 26.73 | 7,126 | 48.90 | 25.13 |
|  |  |  |  |  |  |  |
| Male | 13,694 | 47.34 | 23.69 | 13,694 | 47.34 | 23.69 |
| Female | 23,435 | 65.35 | 27.08 | 13,694 | 50.94 | 23.52 |

**Figure S1. Results from tests of edge weight accuracy**


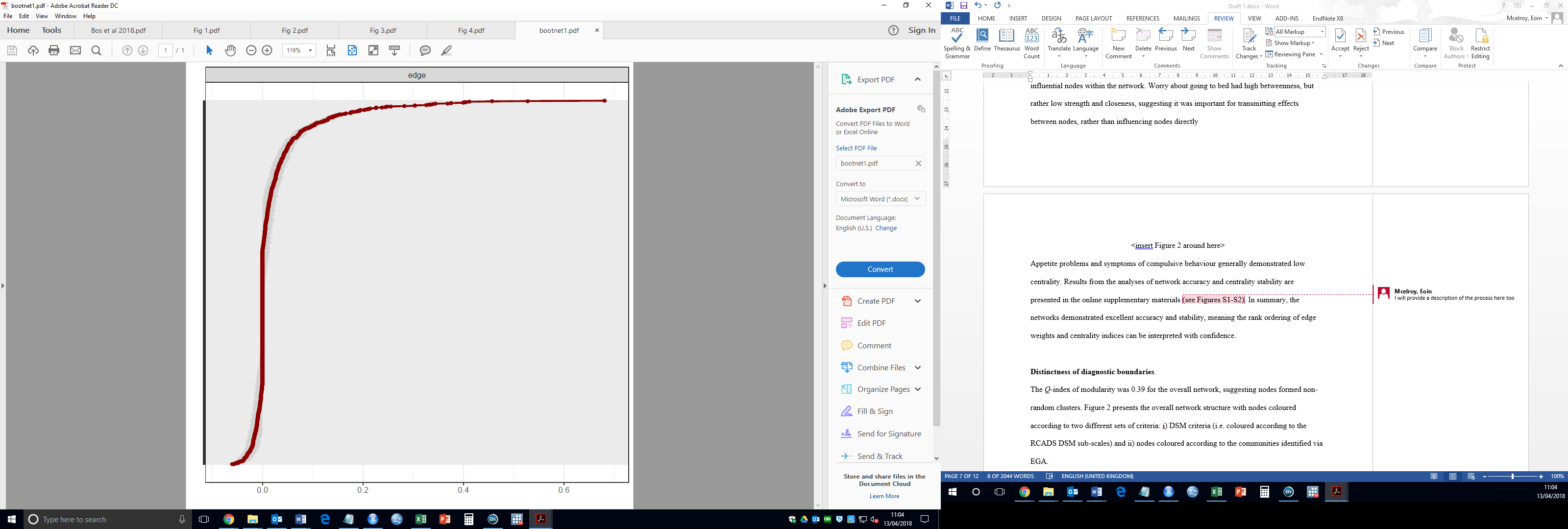


Plot of edge weights and 95% confidence intervals (CIs) calculated using bootstrapping. Extremely narrow CIs, and lack of overlap between CIs indicate that there are significant differences between the strongest edges. As such, the network can be considered highly accurate, and the magnitude and rank ordering of edge weights can be interpreted with confidence. For further details of this test see doi:10.3758/s13428-017-0862-1

**Figure S2. Results from tests of centrality stability**


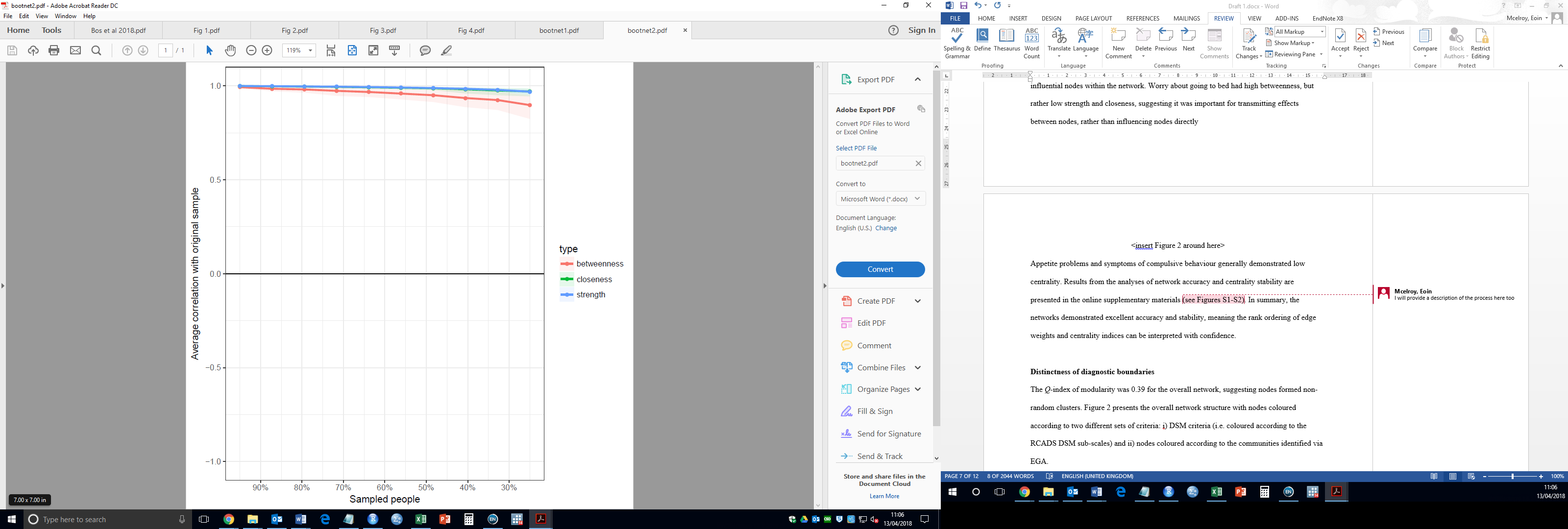


Centrality stability as assessed using the case-dropping bootstrap method. Stability was assessed by re-estimating the network based on increasingly smaller subsets of the original sample The underlying logic of this method is that if the order of centrality estimates from a network based on a small subset is highly correlated to the order of the centrality from the original network, the centrality estimates can be considered stable. In the present analyses, centrality ordering from subsets of as low as 20% were still highly correlated with those of the original sample. This can be quantified as the correlation stability (CS) coefficient, which was 0.75 in the present study (values > 7 reflecting highly stable centrality). These results indicate that the centrality indices were highly stable, and therefore their rank ordeing can be interpreted with confidence. For further details of this test see doi:10.3758/s13428-017-0862-1.

**Figure S3. Networks estimated using alternative methods**

**
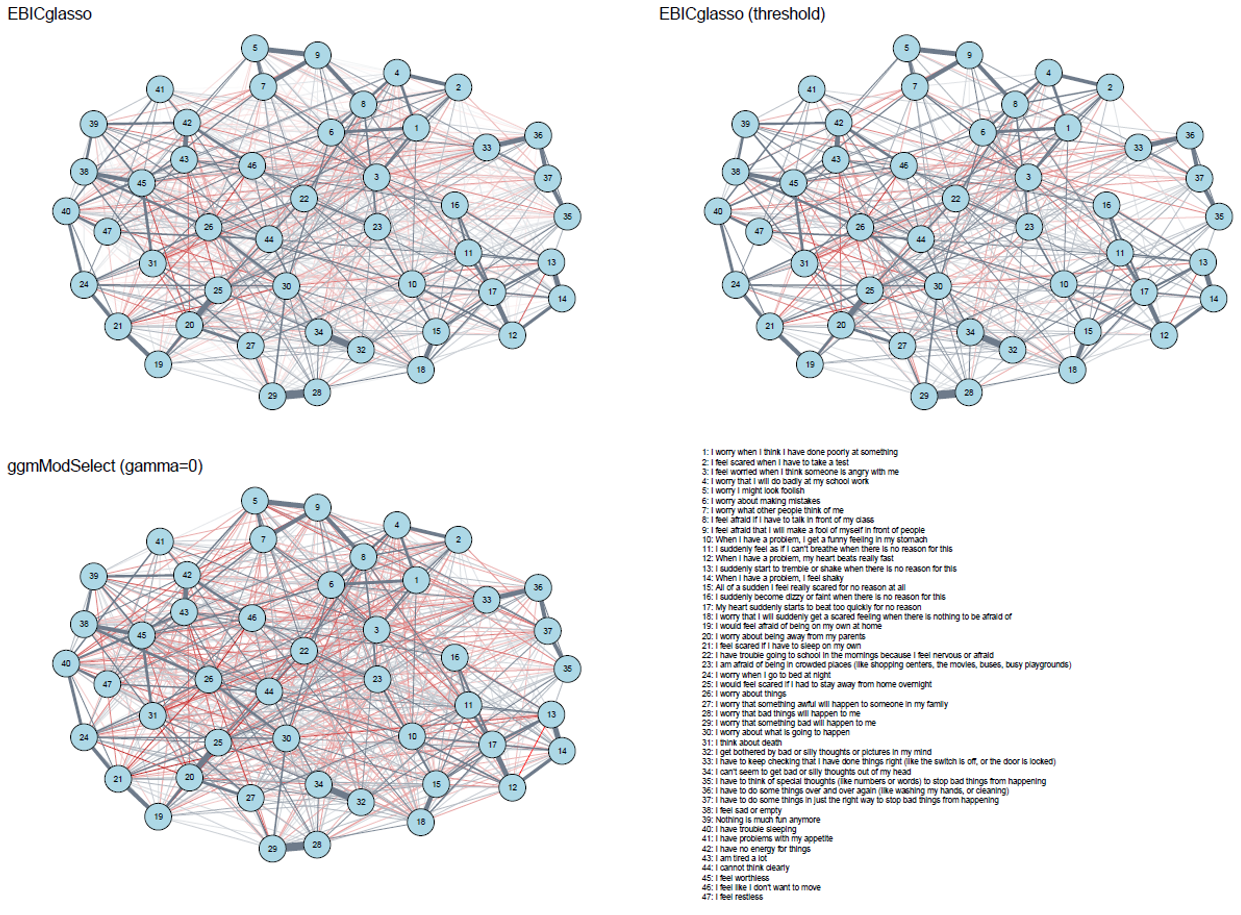
**

**Figure S4. Regularised partial correlation networks for the three age groups**


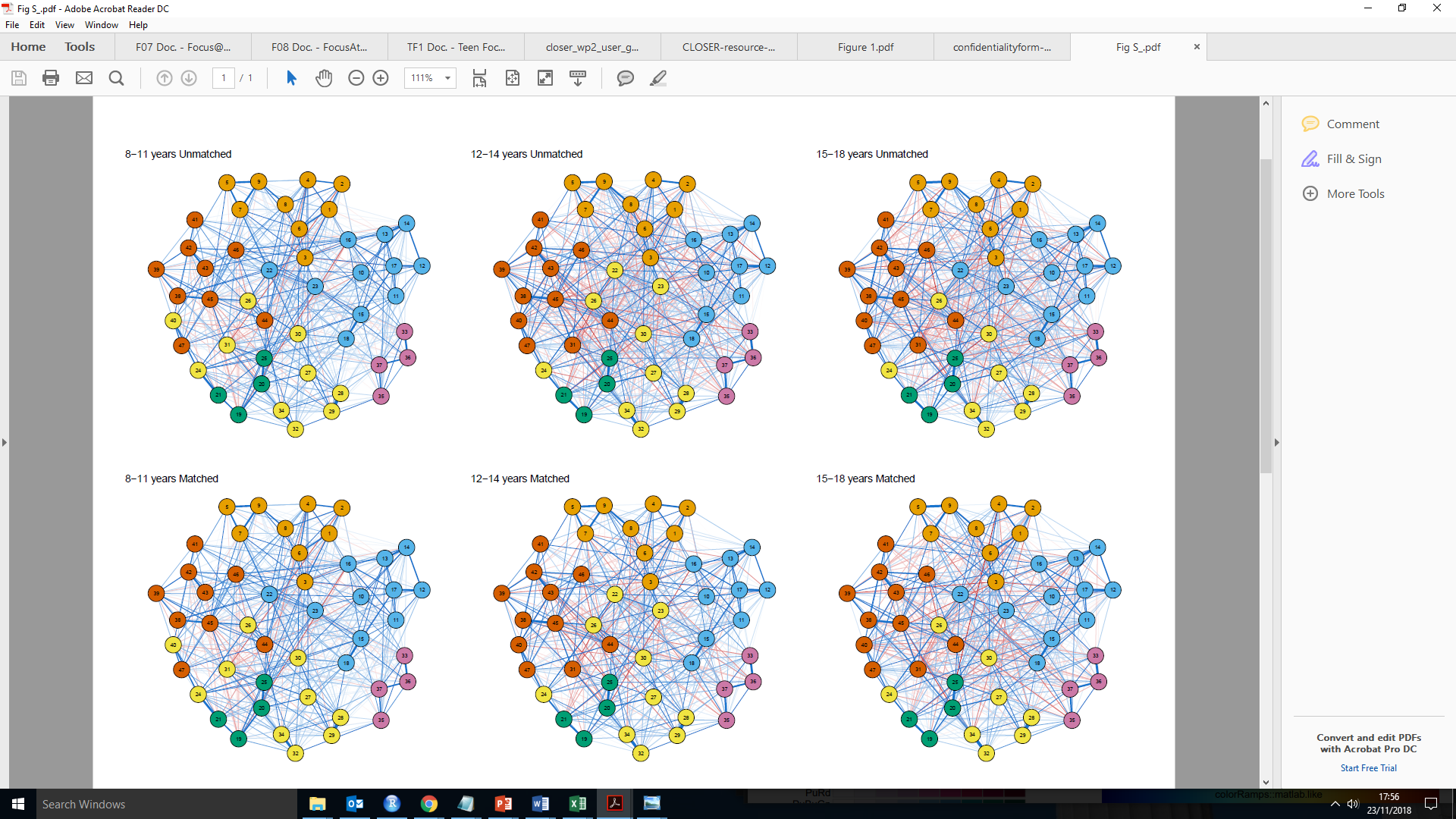


Nodes coloured corresponding to the community structures identified using the walktrap algorithm. Blue lines indicate positive association, red lines indicate negative association.

**Figure S5. Bootstrapped difference tests of strength values by propensity score matched groupings**


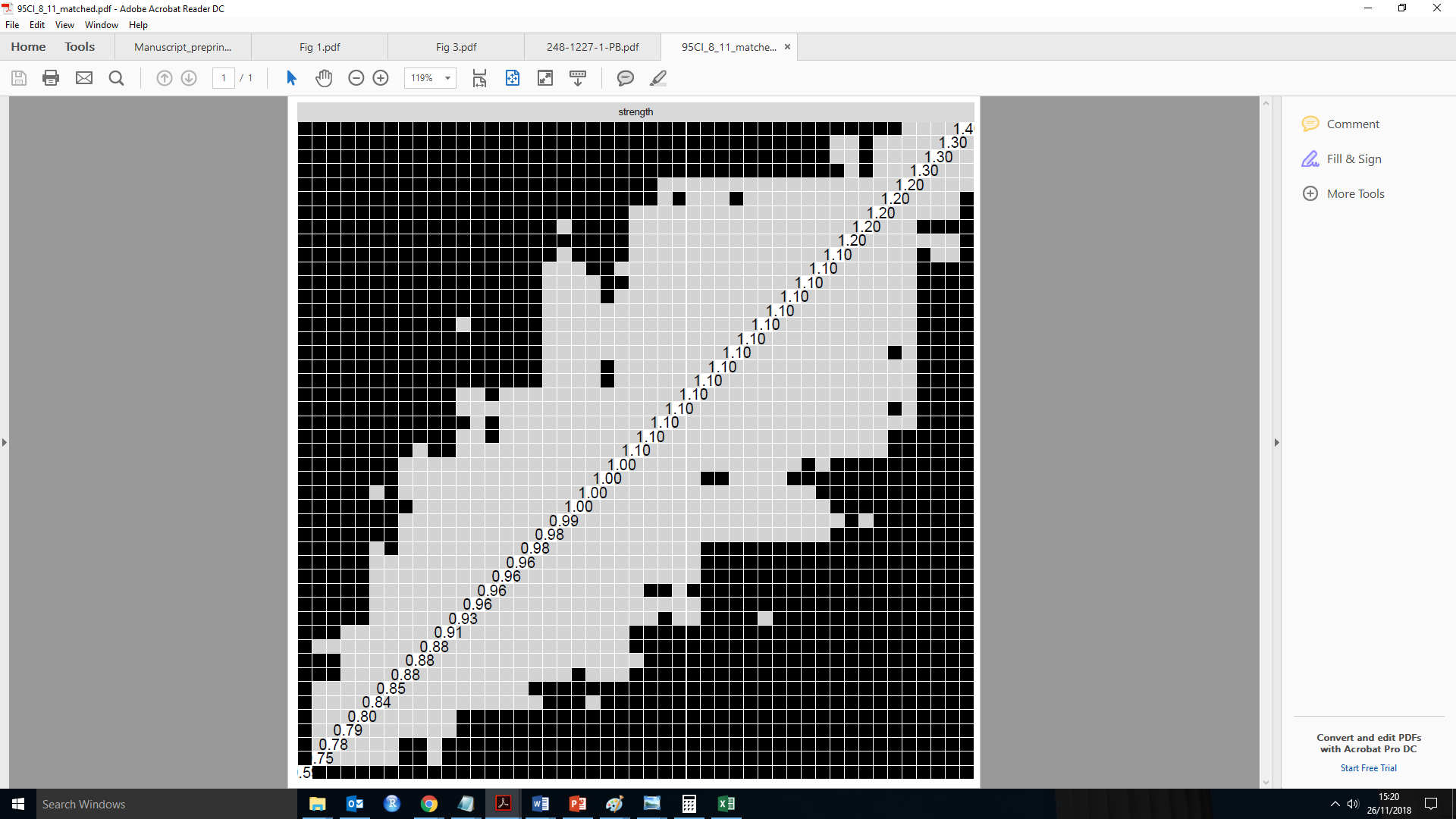


Age 8-11 years


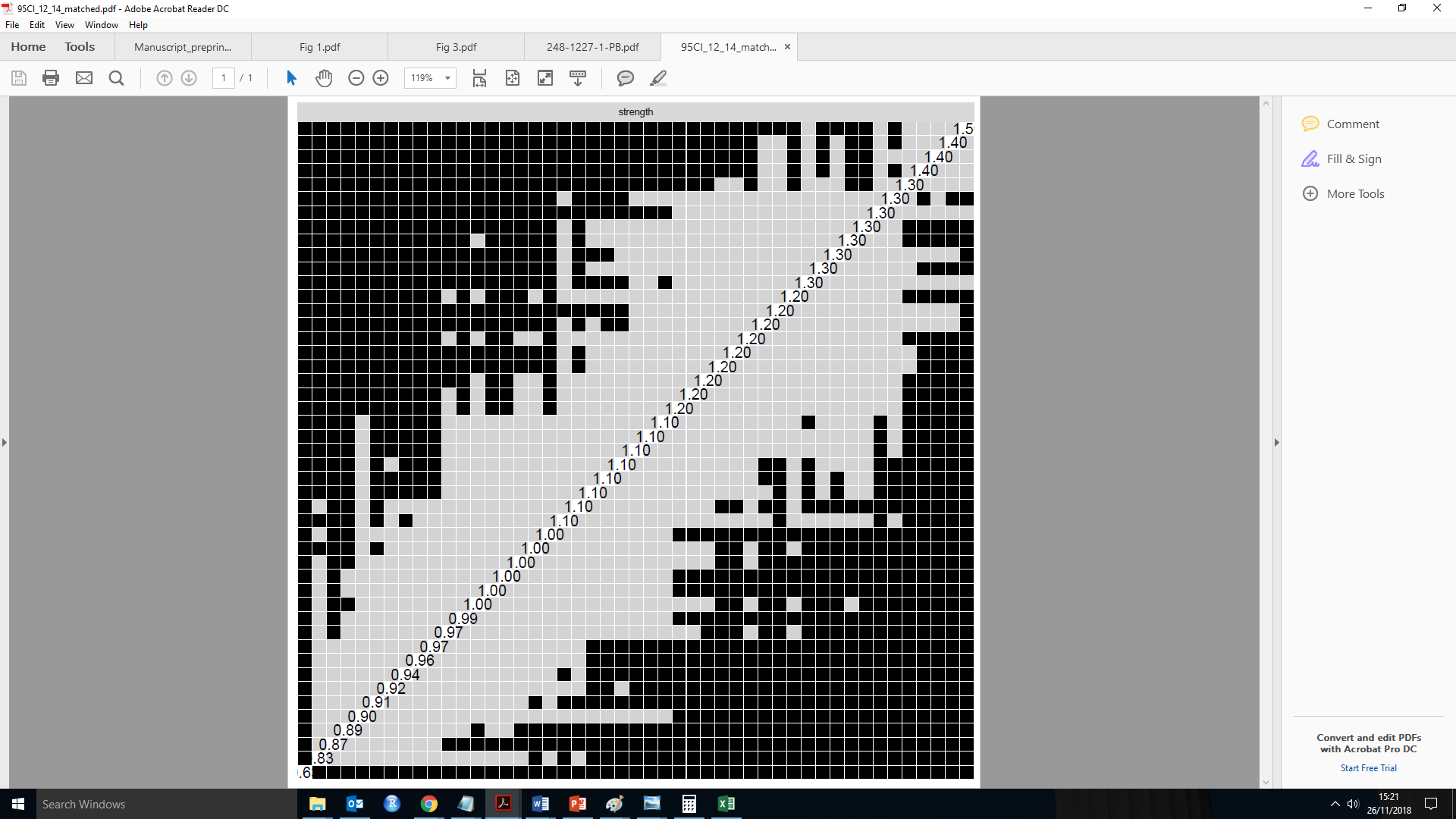


Age 12-14 years


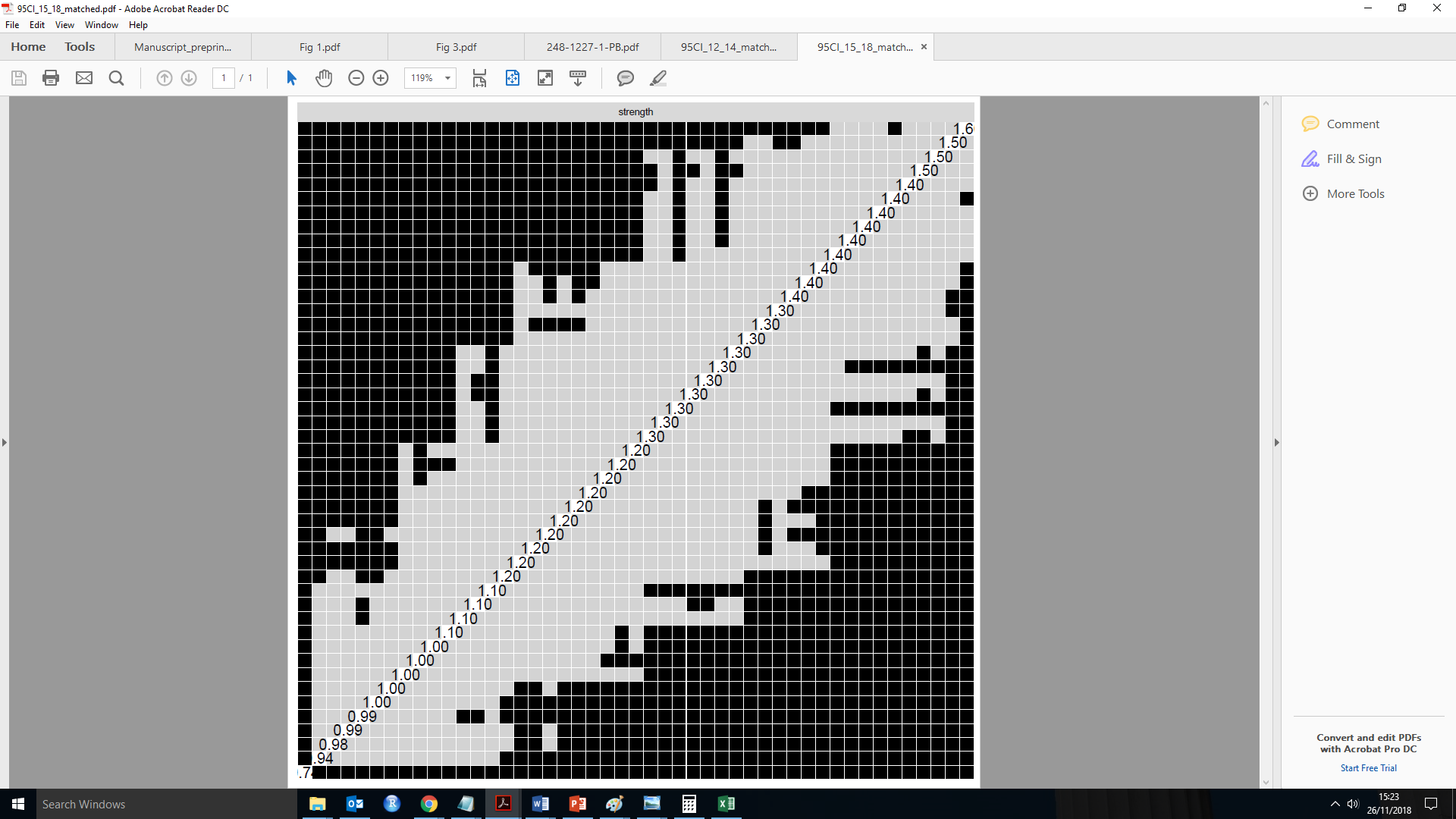


Age 15-18 years


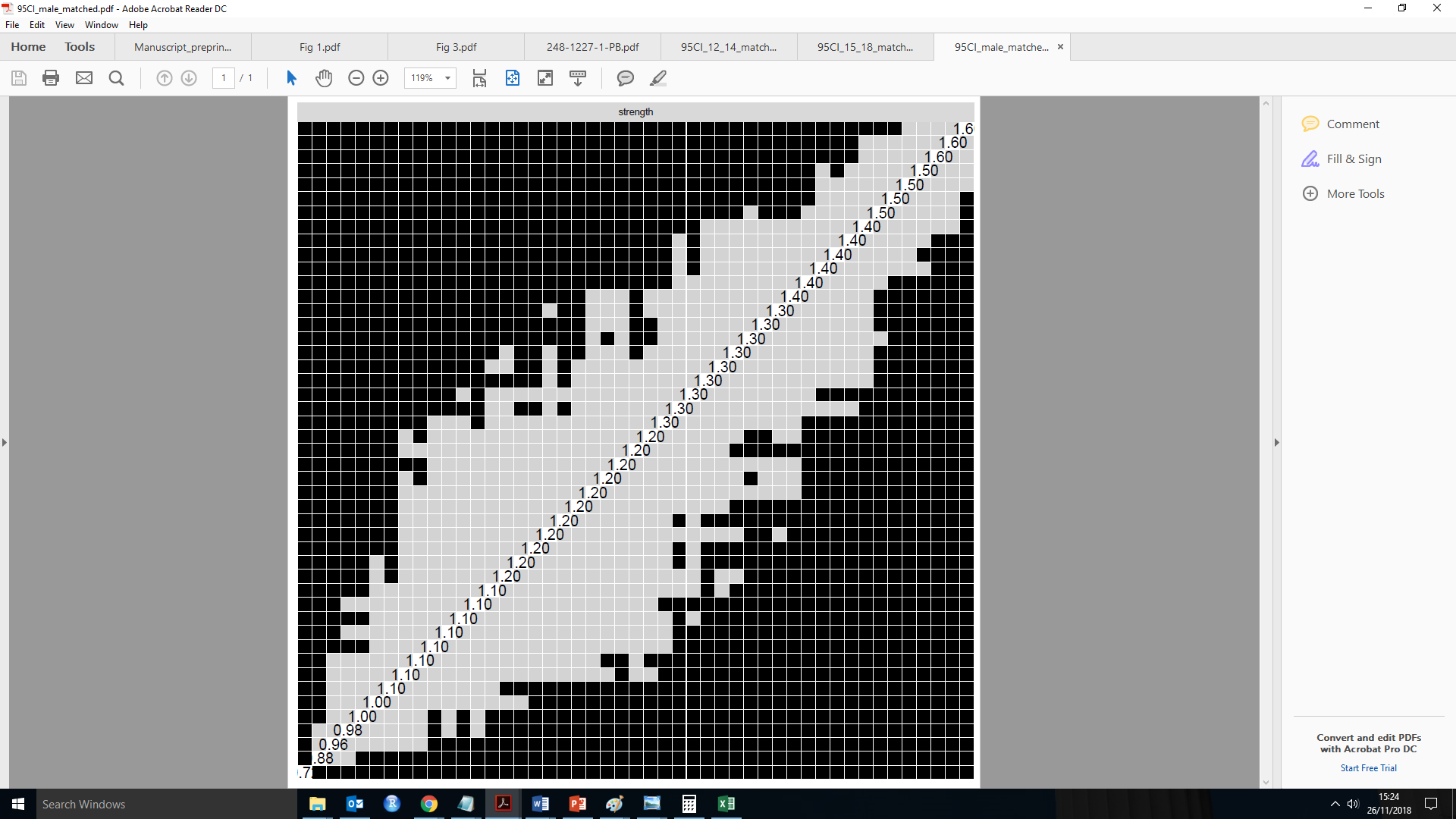

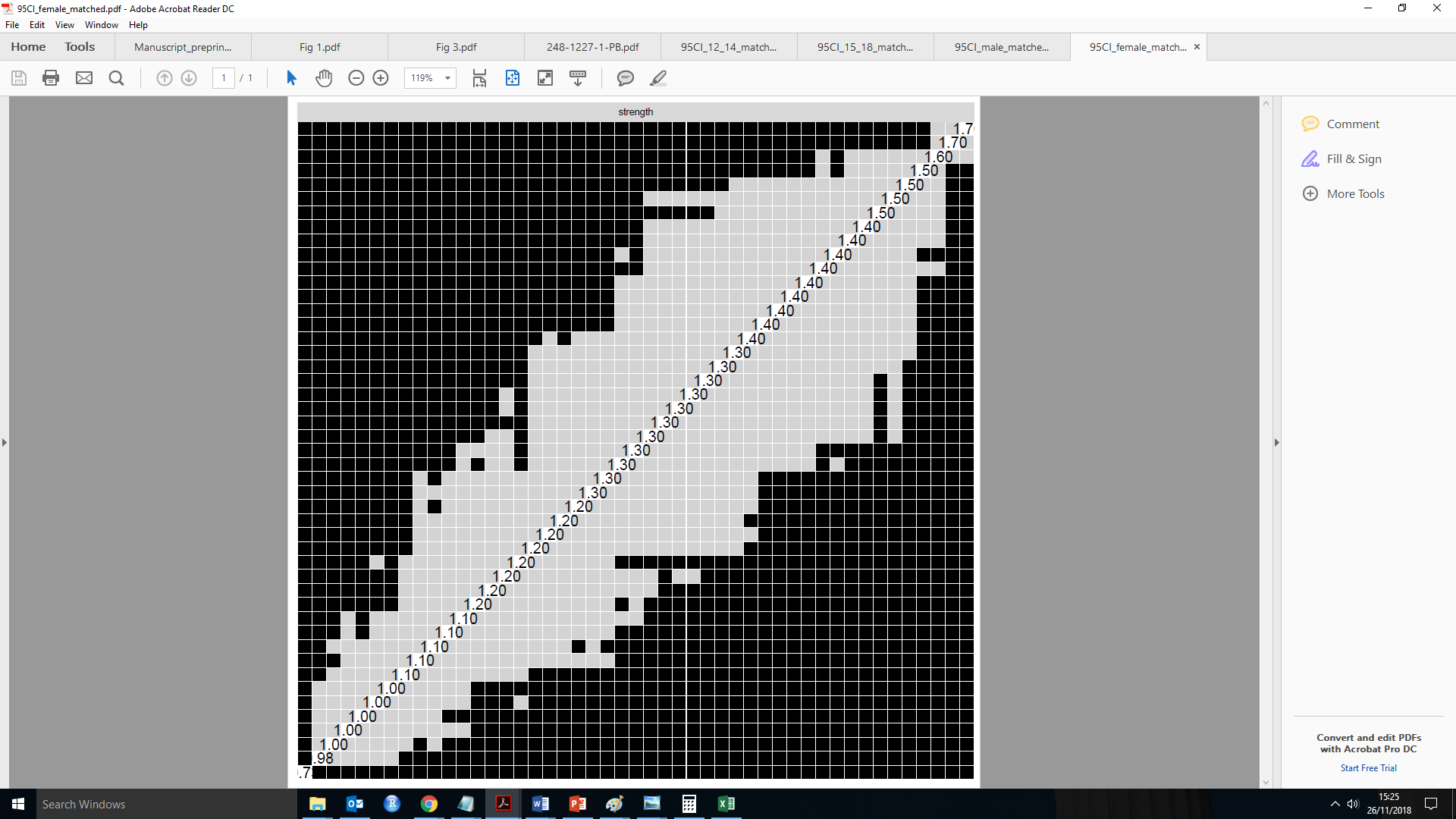


Males

Females

Black boxes indicate non-overlapping bootstrapped 95% confidence interval.

**Figure S6. Networks estimated separately by gender (propensity score matched)**


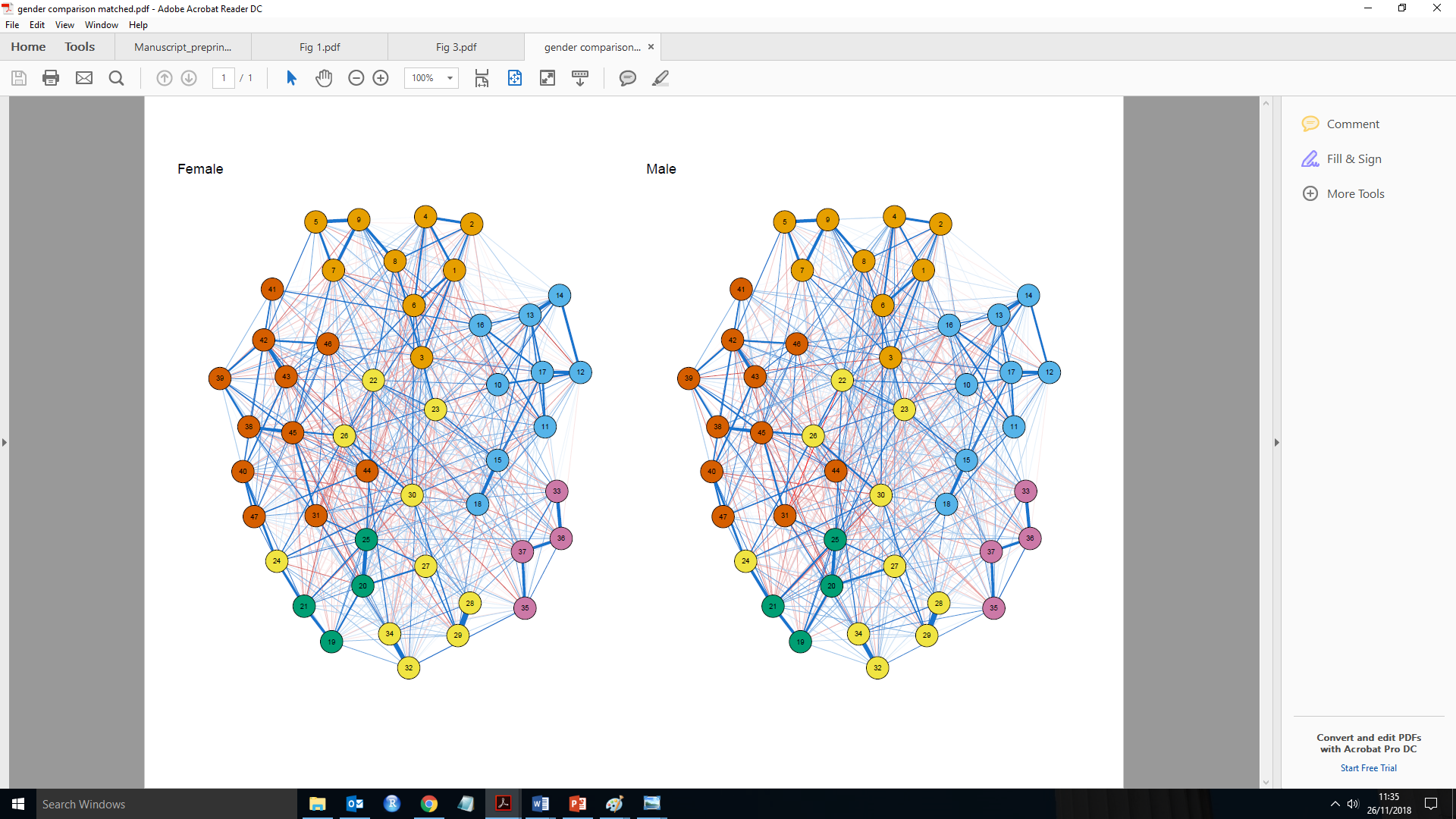


**Figure S7. Centrality values by gender**

**
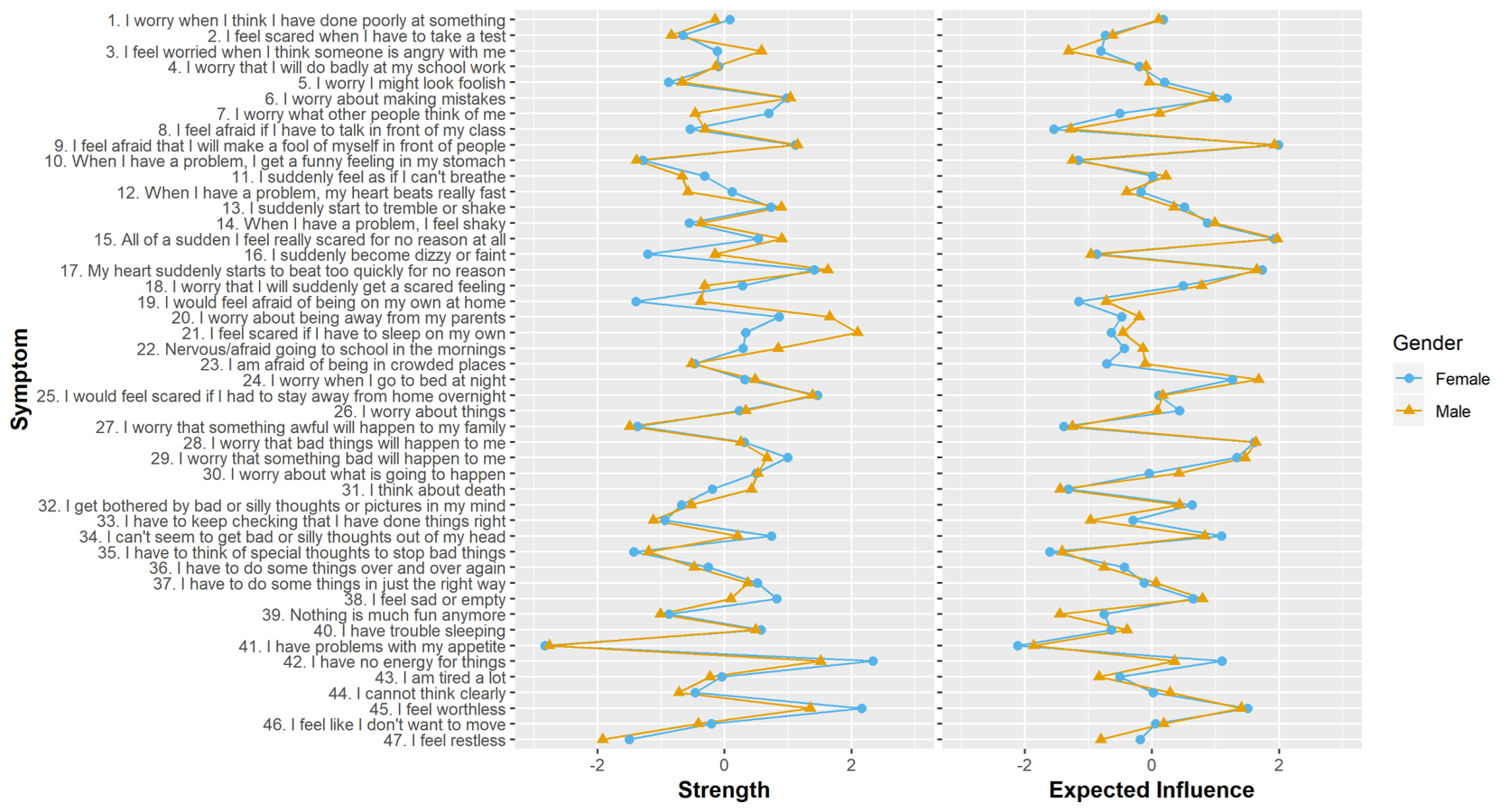
**
